# Non-Invasive Imaging Modalities in Intravesical Murine Models of Bladder Cancer

**DOI:** 10.3390/cancers15082381

**Published:** 2023-04-20

**Authors:** Sydney Relouw, George J. Dugbartey, Alp Sener

**Affiliations:** 1 Matthew Mailing Center for Translational Transplant Studies, London Health Sciences Centre, Western University, London, ON N6A 5A5, Canada; 2 Department of Microbiology and Immunology, Schulich School of Medicine and Dentistry, Western University, London, ON N6A 5C1, Canada; 3 Department of Pharmacology and Toxicology, School of Pharmacy, College of Health Sciences, University of Ghana, Legon, Accra P.O. Box LG 1181, Ghana; 4 Department of Surgery, Division of Urology, London Health Sciences Centre, London, ON N6A 5A5, Canada; 5 Multi-Organ Transplant Program, London Health Sciences Center, London, ON N6A 5A5, Canada

**Keywords:** bladder cancer (BCa), murine model, intravesical, bioluminescence imaging (BLI), magnetic resonance imaging (MRI), micro-ultrasound imaging (MUI), positron emission tomography (PET)

## Abstract

**Simple Summary:**

Bladder cancer (BCa) requires the investigation of alternative therapies. Prior to clinical testing, researchers require the use of animal models to thoroughly investigate their therapeutic efficacy. To appropriately mimic cancer response, the cancer must be developed and treated within the relevant organ. This creates an obstacle with BCa, as cancer presence and progression are difficult to evaluate due to its location. Therefore, non-invasive techniques have been developed that allow for visualization of the cancer from outside the bladder. These techniques include bioluminescence imaging (BLI), micro-ultrasound imaging (MUI), magnetic resonance imaging (MRI), and positron emission tomography (PET). This paper reviews these imaging techniques when used independently or in conjunction in an animal model where BCa is developed within the bladder.

**Abstract:**

Bladder cancer (BCa) is the sixth most prevalent cancer in men and seventeenth most prevalent cancer in women worldwide. Current treatment paradigms have limited therapeutic impact, suggesting an urgent need for the investigation of novel therapies. To best emulate the progression of human BCa, a pre-clinical intravesical murine model is required in conjunction with existing non-invasive imaging modalities to detect and evaluate cancer progression. Non-invasive imaging modalities reduce the number of required experimental models while allowing for longitudinal studies of novel therapies to investigate long-term efficacy. In this review, we discuss the individual and multi-modal use of non-invasive imaging modalities; bioluminescence imaging (BLI), micro-ultrasound imaging (MUI), magnetic resonance imaging (MRI), and positron emission tomography (PET) in BCa evaluation. We also provide an update on the potential and the future directions of imaging modalities in relation to intravesical murine models of BCa.

## 1. Introduction

Bladder cancer (BCa) is the most common genitourinary malignancy and represents the sixth most prevalent cancer in men and seventeenth most prevalent cancer in women, with over 80,000 new cases and 17,000 associated deaths predicted for 2022 in the United States alone [1]. Despite approximately 75% of BCa cases being non-muscle invasive at the time of diagnosis, there is a 78% and 45% 5-year chance of recurrence and progression, respectively [2]. Current standard of care for non-muscle invasive BCa is transurethral surgery followed by Bacillus Calmette–Guerin (BCG) immunotherapy. Although treatment reduces the risk of progression by 30% [3], lifetime surveillance is required, as 40% of patients fail treatment [4] and 70% experience adverse effects [5]. When patients no longer respond to BCG immunotherapy and/or have progressed, and are willing to accept urinary diversion, the next line of treatment is cystectomy (surgical removal of the bladder). Partial or complete cystectomy significantly impacts the quality of life and puts patients at risk for further complications. Considering the current therapeutic impact, BCa has one of the highest lifetime treatment costs per patient compared to other malignancies [6], contributing to the urgent need for the investigation of novel therapies. Murine models of BCa are critical to this investigation as they provide a means for the translational research of potential therapies, allowing for a more comprehensive exploration of in vitro findings prior to clinical testing. Nonetheless, efficacious methods to detect and evaluate BCa response in a pre-clinical model are still being developed.

## 2. Murine Models of BCa

As indicated, the exploration of novel therapies for BCa requires an appropriate experimental model. In vitro models of BCa do not suffice as they are unable to recapitulate human BCa etiology and progression and lack many of the defining features of cancer [7]. In vivo models, on the other hand, have been developed in an attempt to closely replicate the progression, genomic profile, and histology of human BCa [8,9]. Requirements of an ideal murine model of BCa have recently been established as (1) tumors being of urothelial origin to mimic human BCa progression, encapsulating all different stages of the tumor; (2) tumors being able to grow within the bladder to interact with other layers of the bladder wall and for direct exposure to therapeutic agents; (3) tumors being relatively easy to develop within a reasonable time period, making them reproducible and reliable [10,11]. Following these criteria, an intravesical murine model facilitates the opportunity for a successful pre-clinical model to investigate novel BCa therapeutics.

### Categorization of Intravesical Murine Models of BCa

Current intravesical murine models of BCa include carcinogen-induced, orthotopic, transgenic, or humanized mice [12,13,14,15]. Carcinogenic models are developed by the oral administration of chemical carcinogens, which have been reported to recapitulate the progression of human BCa [16,17,18]. However, tumors make take as long as several months to develop using this method [19,20]. Orthotopic models require the inoculation of BCa tumor cells into the bladder wall and can be further categorized as syngeneic or xenogeneic, which consists of the inoculation of mouse or human BCa cells, respectively. Syngeneic models like the carcinogen-induced model allows for the investigation of immune responses. Xenotransplantation, on the other hand, requires the use of immunodeficient mice, eliminating this investigation. Tumor formation in this model is rapid and only takes days to form [21]. Transgenic models of BCa involve the alteration of the mouse genome to investigate individual gene functions [14]. This model is costly and does not mimic the complexity of the microenvironment and interactions during the development of human BCa [22]. Finally, humanized models consisting of mice reconstituted with human immune systems provide a closer replica to humanized immune responses than the previously mentioned models [23,24]. However, this model still fails to mimic the entirety of the human immune system. Nonetheless, intravesical murine models are indispensable to the investigation of BCa etiology and therapeutics.

## 3. Requirement of Non-Invasive Imaging Modalities to Confirm and Evaluate Tumor Progression in an Intravesical Murine Model of BCa

As tumor formation and progression occur within the bladder of intravesical models, BCa is not readily detectable. However, an intravesical model is essential for a clinically relevant investigation of novel therapies. In earlier studies, intravesical BCa tumors were detected and monitored by physical examination such as bladder palpitation or clinical symptoms including hematuria, significant weight loss, and behavioral changes [25,26]. Upon confirmation of such masses or symptoms, it is very likely that the cancer is in its late stages and is likely fatal, making longitudinal investigation of the efficacy of BCa therapies nearly impossible. Another issue that arises during in vivo BCa studies is the need to confirm cancer presence to guarantee balanced randomization of experimental groups. Tumor-take rates for any intravesical model fall short of 100%, thus previous studies required a cohort of animals that would be sacrificed for the purpose of confirming the presence of BCa [27]. However, these few mice do not sufficiently represent what is occurring in the remainder of the experimental group. Thus, accurate, non-invasive assessment of BCa tumors has become a primary objective for developing intravesical murine models.

Established modalities that reveal and evaluate the occurrence of BCa within murine models include bioluminescence imaging (BLI), micro-ultrasound imaging (MUI), magnetic resonance imaging (MRI), and positron emission tomography (PET), as summarized in Table 1. These modalities have provided a better understanding and visualization of cancer development [28], early detection [29], and therapeutic efficacy [30,31,32] in cancers such as breast [29,31,32], prostate [33], colon [28], and lung [30]. BLI relies on the biochemical generation of light for tumor assessment while PET utilizes radiotracers to investigate tumor burden while also providing information on the metabolic and biochemical functions. MUI and MRI produce 2-dimensional and 3-dimensional images of internal structures such as tumors, respectively. Substances such as contrast agents have been employed to provide more detailed images. Due to the non-invasiveness of these modalities, they have allowed for the initial and repeated assessment of tumor burden in all experimental animals, providing longitudinal assessment and reducing the number of required animals. This makes them an integral part of the translational research in oncology. However, these methods have required further revision for investigation within murine models of BCa. Therefore, the remainder of this review will focus on recent advancements within imaging modalities in intravesical murine models of BCa.

## 4. Non-Invasive Imaging Modalities

### 4.1. Bioluminescence Imaging (BLI)

**Figure 1:**
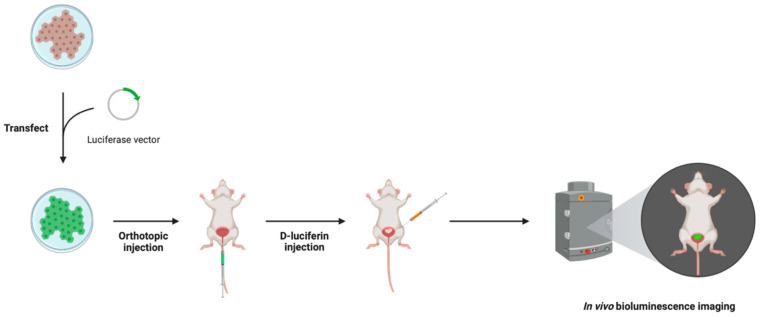
Bioluminescence imaging (BLI) method in an intravesical murine model of BCa, where a BCa cell line is transfected with a luciferase vector that is subsequently inoculated into the bladder wall of the murine model. The D-luciferin substrate is administered via intravascular or intraperitoneal injection 10–18 min prior to visualization to stimulate the reporter system. Visualization occurs using an in vivo imaging system that detects the emission of green light when luciferase converts D-luciferin to oxyluciferin. Figure prepared with BioRender (biorender.com, accessed on 1 February 2023).

BLI allows for the visualization of BCa cells through the detection of light emission using an in vivo imaging system. BCa cell lines are first transfected with a luciferase vector and then inoculated into the bladder wall of a murine model. Subsequent intravascular or intraperitoneal administration with a D-luciferin substrate stimulates its oxidation by luciferase, converting D-luciferin to oxyluciferin, resulting in the emission of green light at 550–570 nm (Figure 1). Importantly, this reporter system is oxygen- and ATP-dependent. Therefore, only metabolically active cancer cells are capable of producing light. The optimal time to wait after D-luciferin administration for BCa visualization ranges from 10 to 18 min [10,34,35,37,38]. 

As genetic engineering of the cells is required, this imaging modality is limited to an orthotopic BCa model. In studies detecting intravesical BCa tumor presence in vivo, BLI confirmed tumor presence as early as 4 days post-inoculation [39] (Table 1). Other studies detected tumor presence at 5 to 15 days [35,36,37,41,51,52,53] while another was as late as 33 days [37]. This discrepancy may be due to the sensitivity of the BLI system or the number of living cells that remained after inoculation. Whether the inoculated cells were human or mouse-derived did not appear to affect the detection time, however, the mouse strain did. For example, one study utilizing mice with severe combined immunodeficiency (SCID; lacking both T and B lymphocytes) visualized bioluminescence on day 4 [51], whereas another study utilizing the double mutant, SCID-beige, did not visualize bioluminescence until 33.9 ± 18.3 days [37]. Both studies utilized the same cell line and the latter study inoculated more than double the number of cells than the former study, ruling out the cell line and inoculated cell number as potential causes of this discrepancy. Interestingly, no clinical signs of BCa were reported at any of these time points. Only one study reported a false negative with one out of 12 mice possessing a small tumor cell nest that did not express bioluminescence, but was confirmed by histology and immunohistochemistry at necropsy [36].

Moreover, the quantity of orthotopically implanted luciferase-expressing human BCa cells has been shown to positively correlate with bioluminescence intensity [10]. This can be attributable to the increase in metabolically active cells and thus higher ATP production. Cell line-dependent coefficients of determination (R^2^) ranged from 0.95 to 0.99, which was corroborated by another study [34]. In the same study, the authors utilized ultrasound to demonstrate a positive correlation between the tumor size and bioluminescence, with an R^2^ value of 0.97 ± 0.02. This finding was also corroborated by another study [36]. In a preceding study, using an orthotopic xenograft model, the authors reported varying tumor size and bioluminescence intensity, which correlated overtime with R^2^ values ranging from 0.75 to 0.92 [39]. However, this was followed by a decline in bioluminescence, alluding to a limitation of BLI (Table 1). The same research group also observed that bioluminescence correlated differently to tumor size based on the xenograft type, attributing this to the presence or absence of hypoxia and necrosis [34]. Several studies have also demonstrated a loss of bioluminescence strength, with BCa progression. For instance, Scheepbouwer et al. [38] observed an increase in bioluminescence from days 5 to 7 to days 18–29 followed by a plateau until days 29–31, whereafter the mice were sacrificed. Investigation of the stability of long-term, orthotopic implantation with BLI demonstrated positive but relatively decreased activity over a 2-month period [35].

Interestingly, the photo emission intensity has also been demonstrated to be lower in murine models compared to in vitro models [10]. This disparity was attributed to interference of the abdominal wall. This is supported by preceding studies where the absorption of light by tissue, dark fur, pigmented skin, and BCa stage have also been indicated as additional potential causes, initiating further investigation [34,36]. These interferences may contribute to the inability to detect cancer cells immediately after inoculation. As previously mentioned, Black et al. [34] investigated the role of hypoxia and necrosis in the relationship between bioluminescence and tumor size using 253J-BV and KU7 xenografts; cell lines now discovered to be cross contaminated with HeLa. Nonetheless, KU7 tumors were found to have substantially more hypoxic and necrotic tissue compared to 253J-BV tumors, contributing to the R^2^ values of 0.39 and 0.90 for bioluminescence and tumor burden, respectively. They speculated that the loss of vascular differentiation in the KU7 tumors may have resulted in the inadequate delivery of luciferin to the entire tumor. In a subsequent study, Jurczok et al. [36] denoted the loss of bioluminescence in large tumors to be due to necrosis and hemorrhage, confirmed by histological analysis, which contributed to the large tumor volume but lower bioluminescence. As further confirmation, immunohistochemical staining demonstrated substantially lower levels of luciferase in these areas compared to vital tumor areas. In conclusion, BLI is a reliable modality to confirm and quantitively evaluate early-stage BCa presence, but can only qualitatively assess the BCa presence in advanced stages.

### 4.2. Micro-Ultrasound Imaging (MUI)

**Figure 2:**
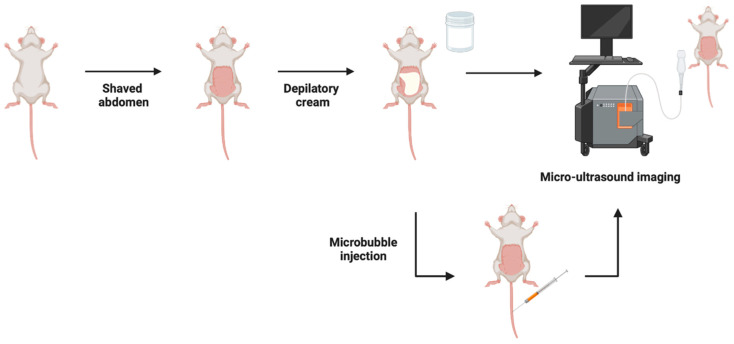
Micro-ultrasound imaging (MUI) method in an intravesical model of BCa where the abdomen is shaved, depilatory cream is applied to remove fine hairs, and high viscosity ultrasound gel is utilized during imaging to enhance the image resolution. Subsequent studies administered microbubbles via intravenous injection prior to imaging for visualization and quantification of the superficial tumor vasculature. Figure prepared with BioRender (biorender.com, accessed on 1 February 2023).

MUI utilizes soundwaves to produce 2-dimensional images of organs and other tissues. Compared to conventional ultrasound, which uses a frequency of 2–10 MHz, MUI uses shorter spatial pulses, resulting in a mean frequency of 40 MHz, higher resolution, and higher clarity images. For example, resolutions of 80 μm in the lateral plane and 40 μm in the axial plane were achieved [27,40,43], whereas others have achieved a 30 μm resolution [10]. To enhance the image quality in a murine model, the abdomen is shaved, depilatory cream is used to remove fine hairs, and a high viscosity ultrasound gel is applied (Figure 2). These modifications allow for imaging of both superficial and invasive tumors where the tumor volume can be calculated using MUI with the formula:

π/6 × length × width^2^ (1)

MUI has a short acquisition time of approximately 5 min per mouse [43], further underscoring its attractiveness for non-invasive imaging (Table 1). Furthermore, MUI is independent of tumor origin, whether orthotopically, carcinogen, or genetically induced, making it suitable for use in conjunction with all intravesical BCa models. However, as summarized in Table 1, reported disadvantages of MUI include the lack of 3-dimensional imaging [42] and a reliance on a skilled operator to detect BCa tumors as well as the restricted availability of this technology [42,44].

The mean BCa tumor detection time in a syngeneic orthotopic model using MUI was found to be 10 days, ranging from 8 to 17 days, whereas that for the clinical symptoms of BCa was 20.8 days, ranging from 14 to 28 days [21,40,43]. Subsequent studies were able to detect orthotopic BCa tumors as early as 4 [39], 7 [47] and 11 days [38,40,48], which did not appear to be influenced by the cell line or mouse strain. Conversely, a more recent study was unable to detect tumors in a syngeneic orthotopic model until the thirteenth day and mice began to die on the sixteenth day from clinical symptoms [44].

A MUI validation study imaged a syngeneic orthotopic model before and after BCa cell inoculation [43]. At each imaging session, mice were sacrificed to confirm tumor presence or absence, as indicated by MUI. The tumor detection rate was 87%, with 13 out of 15 mice being correctly identified as having BCa (Table 1). All mice possessed non-muscle invasive BCa and only one mouse developed clinical symptoms prior to tumor detection. Moreover, stereochemistry was used to confirm the tumor location and volume, with the latter producing a correlation coefficient of 0.97. The smallest tumor to be detected by MUI in this study was approximately 0.52 mm^3^. Interestingly, Scheepbouwer et al. [38] detected tumors as small as 0.4 mm^3^ (Table 1), while Chan et al. [40] detected tumors between 1 and 3 mm in diameter, further underscoring the sensitivity of MUI.

Further refinement of MUI includes the use of ultrasound contrast agents. Microbubbles, or nanobubbles of gas, are administered intravenously and expand and compress in response to ultrasonic waves, enhancing contrast and allowing for the visualization and quantification of superficial tumor vasculature (Figure 2). Chan et al. [40] utilized this method to effectively target the expression of vascular endothelial growth factor receptor 2 (VEGFR2), a regulator of endothelial migration and proliferation, in a syngeneic orthotopic model. In addition to the modifications mentioned previously, an elastic band was applied over the lower abdomen to prevent respiration and bowel movements from creating artifacts but without inhibiting blood flow. This study reported the performance time of high-contrast MUI to last approximately 60 min per mouse, which is a substantial change compared to MUI alone (Table 1). A subsequent study successfully used contrast-enhanced ultrasound to visualize the perfusion status of an orthotopic xenograft model [39]. An elastic was also placed over the abdomen, mimicking the previously mentioned study. Many advancements have been made to ultrasound imaging, contributing to its success as a rapid, multiuse, non-invasive imaging modality for intravesical BCa in murine models.

### 4.3. Magnetic Resonance Imaging (MRI)

MRI produces high-resolution 3-dimensional images of anatomical structures via a strong magnetic field and radio waves. Several studies have investigated the feasibility of MRI as a method for the identification and quantitative analysis of intravesical murine BCa tumors. In a carcinogen induced BCa murine model, MRI was used to calculate the bladder wall and tumor area [42] (Table 1). In this study, MRI produced T1 and T2 weighted images with ~100 μm spatial resolution in less than 10 min per mouse (Table 1). Bladder wall measurements, representative of tumor burden, were calculated by subtracting the area of inner lumen from the area of the outer edge of the bladder using single axial images. The area of the bladder wall was found to be strongly associated with tumor stage (*p* = 0.0003) despite being weakly correlated with ex vivo bladder weight (r_s_ = 0.39). Interestingly, the bladder wall area was also strongly associated with both non-muscle invasive and muscle invasive diseases, whether grouped as individual stages (*p* = 0.003) or invasiveness (*p* = 0.002). An acknowledged limitation of this study was the use of one axial image in the calculation of the bladder wall area. Multiple sections are recommended for a more complex analysis. However, the authors claimed that one image was sufficient in demonstrating the usefulness of MRI quantification and the ease of image processing and analysis. A subsequent study also revealed a significant positive correlation between tumor diameter, as determined by MRI, and tumor stage [45]. As further evidence for the usefulness of MRI as a tumor quantification method, Black et al. [34] found the correlations of MRI and autopsy for tumor volume and weight to be 0.96 and 0.92, respectively, in an orthotopic xenograft model (Table 1).

To enhance the MRI acquisition capabilities, Kikuchi et al. [45] investigated various contrast agents and their ability to optimize contrast to noise in a syngeneic orthotopic model. They found that the intravesical instillation of 50 [mu]L Gd-diethylenetetramine pentaacetic acid with 50 [mu]L air prior to imaging, compared to air, undiluted and diluted Gd-diethylenetetramine pentaacetic acid alone, resulted in better delineation of tumors from the bladder wall. In the same study, the authors imaged mice on days 10, 14, 17, and 24 to investigate the MRI tumor detection capabilities. MRI produced T1-weighted images with a 1.5 mm thickness [45]. On day 10, 14 tumors were identified by MRI whereas 17 tumors (all non-muscle invasive) were identified by pathology. The false negatives were attributed to the tumor sizes being less than 1 mm in diameter. On day 14, nine tumors were identified by MRI, however, two of these abnormalities were confirmed to be hyperplasia and catheter-induced trauma during BCa cell implantation [45]. By day 14, tumor detection rate by MRI was 86.4% (Table 1). Prior to this point, no clinical signs were detectable in any of the mice, demonstrating the ability of MRI to detect early-stage BCa. On day 17, MRI imaged all bladders to be tumor-filled, while the MRI identified tumors invading the bladder muscle layer on day 24, which was later confirmed by histology [45]. In addition to tumor detection, the accuracy of MRI for determining tumor size was also investigated. MRI-based measurements strongly correlated with the actual tumor size (r^2^ = 0.977, *p* < 0.001). The smallest tumor size detected was 1.5 mm in diameter, which they attributed to the use of a second set of transverse images shifted by 1 mm (Table 1). This study demonstrates the ability to accurately detect and quantify both early and late-stage BCa in an optimized MRI approach [45].

Further advancements in MRI for BCa assessment include the use of mesoporous silica nanoparticles (MSNs) in conjunction with fluorescence [53]. Sweeney et al. [53], attracted to the non-toxicity and tolerability of MSNs, covalently bound fluorescein molecules to MSNs and injected them into a syngeneic orthotopic model. Compared to non-labelled tumors, MSN-labelled BCa tumors displayed more intricate features, which were later confirmed by histology to be areas of faster tumor growth and high cell density. This study demonstrates the combination of MRI and MSNs to provide more detailed images of tumors, allowing for the detection of defining BCa features.

### 4.4. Positron Emission Tomography (PET)

PET, dissimilar to the modalities described above, evaluates the metabolic activity of organs and tissues, providing information on the physiology and anatomy in addition to quantitative cancer detection. Molecules naturally used by the organs or tissues of interest are tagged by a radioactive atom, producing a radiotracer, allowing for the detection and evaluation of diseases such as cancer (Table 1). Molecular targets for imaging cancers are derived from pathways or proteins typically overexpressed in cancer cells or tumors. As such, a diverse selection of radiotracers exists, giving PET its high specificity. Current radiotracers in cancer research include ^68^Ga-fibroblast activation protein inhibitor (FAPI), ^68^Ga-ligand-prostate specific membrane antigen (PSMA), ^18^F- and ^11^C-choline and ^11^C-methionine, for example. The most widely used radiotracer is 2-^18^F-fluoro-2-deoxy-glucose (^18^F-FDG). ^18^F-FDG is a glucose analog and works based on the premise that cancer cells, which are more metabolically active than non-cancerous cells, will uptake glucose at a higher rate. This uptake is detected by PET, thus identifying cancerous regions. The attractiveness of PET is attributed to its ability to identify genomic aberrations and the dysregulation of proteins [46], which cannot be detected by the previously described imaging modalities. Unfortunately, the evaluation of BCa with PET is difficult due to the accumulation of ^18^F-FDG within the bladder from renal excretion, which obstructs the delineation of the tumor from the bladder wall and hinders the detection of BCa [49] (Table 1).

This limitation currently makes PET an unattractive option for BCa imaging. However, its unique ability to detect phenotypic changes has encouraged several studies to investigate modifications to potentially improve detection in intravesical murine BCa models. For example, Mahendra et al. [50] investigated the efficacy of two isomers of ^18^F-fluoro-alpha-metylphenylalanine (^18^F-FAMP), L-2-^18^F-FAMP, and D-2-^18^F-FAMP in an orthotopic xenograft model. Following one hour of intravenous injection, the biodistributions of both radiotracers demonstrated an accumulation in BCa tumors [50]. L-2-^18^F-FAMP demonstrated significant accumulation compared to ^18^F-FDG, whereas D-2-^18^F-FAMP showed a noticeably higher but non-significant accumulation compared to ^18^F-FDG. PET imaging at 1 h post-injection also demonstrated a clear visualization of all three radiotracers. Interestingly, at 3 h post-injection, the visualization of tumors was clearer, which was attributed to their fast elimination rate including rapid blood clearance and low renal accumulation [50].

Concurrently, Pereira et al. [47] investigated the efficacy of using galectin-targeted imaging in an orthotopic xenograft model. Following one hour of the intravesical administration of ^18^F-labeled galactodendritic unit 4, accumulation was significantly higher in BCa tumor-bearing mice compared to non-tumor bearing mice, with SUV_mean_ values of 43.5 ± 4.2 and 2.0 ± 0.4, respectively [47]. These findings demonstrate the selectivity of galectin-targeted radiotracers in imaging intravesical BCa cells. Nevertheless, this study utilized a high galectin-expressing cell line and acknowledged the need for the further investigation of the nonspecific binding of the radiotracer. Neither study utilized PET to confirm the presence of BCa before commencement, rather, bladder palpation and MUI were used, respectively. Thus, PET has been demonstrated to be potentially useful to monitor BCa tumors, only after detection by an alternative modality.

**Figure 3:**
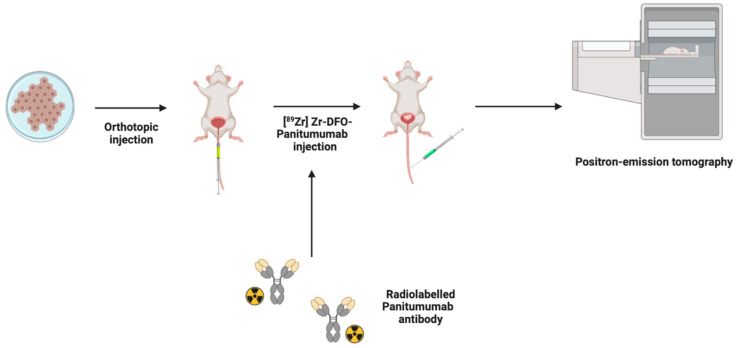
Positron emission tomography (PET) method in an intravesical murine model of BCa in which EGFR-expressing BCa cells are inoculated into the mouse bladder wall. Antibodies are conjugated and radiolabeled producing [^89^Zr] Zr DFO-panitumumab, which was administered intravenously, and mice were imaged 72 h later. Figure prepared with BioRender (biorender.com, accessed on 1 February 2023).

Immuno-PET is another branch of PET imaging that utilizes antibodies for the specific targeting of molecules expressed by cancer cells and tumors. In an orthotopic xenograft model, detection of the epidermal growth factor receptor (EGFR)-expressing BCa cells by the radioimmunoconjugate [^89^Zr] Zr-DFO-panitumumab was investigated [48] (Figure 3). Imaging after 72 h after intravenous administration, [^89^Zr] Zr-DFO-panitumumab injection resulted in tumor size-dependent accumulation, with a correlation coefficient of 0.99 exhibiting the quantitative capability of PET. Interestingly, these mice demonstrated inconsistencies in their EGFR protein expression and tumor sizes [48]. Thus, it is speculated that the strong correlation is caused by the non-specific binding of the antibody due to enhanced permeability and retention effects.

Although imaging with PET in intravesical BCa models is limited due to the high renal excretion activity, studies have continued to investigate promising solutions to apply the advantages of PET to the detection and evaluation of BCa.

### 4.5. Imaging Modalities in Combination for BCa

Importantly, the validation of the use of these imaging modalities has also been of great interest. To do so, studies have compared the ability of these imaging modalities to detect and assess intravesical BCa in murine models by assessing the correlations of detection of BCa between two or more imaging modalities. For example, Black et al. [34] investigated the correlation between BLI and MRI on tumor volume using an orthotopic xenograft model. On week 4 of their study, a correlation coefficient of 0.93 was found within the 253J B-V tumors, suggesting the two imaging modalities produced comparable values. They also used KU7 tumors, where they did not observe a strong correlation at any timepoint throughout the study [34]. However, as previously mentioned, this cell line has been found to be contaminated, thus disproving its use in BCa research. Another study conducted quantitative comparisons between BLI and MRI in an orthotopic xenograft model [37]. Although both MRI and BLI detected tumors at day 6, they reported that MRI was more difficult to capture than BLI due to the movement of the surrounding organs. This resulted in false negatives by MRI, which were confirmed to be BCa by BLI [37]. Nonetheless, the authors suggested that although BLI was more suitable for routine measurements, MRI was able to provide more information such as tumor size and location. Seo et al. [11] also utilized MRI to successfully verify the qualitative and quantitative analysis of tumor growth in response to the downregulation of syngeneic orthotopic tumors using BLI.

Moreover, Jager et al. [39] demonstrated a positive correlation between BLI and tumor volume determined by MUI in an orthotopic xenograft model. UM-UC1, UM-UC3, and UM-UC13 tumors had correlation coefficients of 0.76, 0.82, and 0.93, respectively, despite a reported gradual loss by BLI. Raven et al. [10] further demonstrated this correlation in an orthotopic xenograft model, with a correlation coefficient of 0.97 ± 0.02. These findings suggest that multi-modal use of MUI can make up for BLI’s inability to directly provide information on the tumor size.

Scheepobouwer et al. [38] also investigated the use of BLI in conjunction with two variations of ultrasound, high resolution ultrasound and photo-acoustic imaging (PAI), which use optical properties of tissues to provide detailed images of vasculature [54]. A 4-week study was conducted using an orthotopic syngeneic model. BLI, as discussed earlier, demonstrated an initial increase followed by a plateau and then a decrease in signal, whereas ultrasound demonstrated a continuous increase in tumor volume over time, and PAI demonstrated an increase in oxygen saturation 13 days after inoculation, followed by a gradual decrease until the end of the study [54]. Furthermore, BLI was able to detect tumors as early as 5–7 days, demonstrating its high sensitivity, whereas ultrasound was only able to detect tumors at day 11, which was when PAI began. However, ultrasound and PAI were able to provide information on irregular tumor growth and functionality, unlike BLI. Together, these studies suggest that a multi-modal approach can be utilized when non-invasively imaging intravesical BCa for better detection and evaluation.

## 5. Urinary Analysis for BCa Detection and Progression

As discussed, common limitations of the above imaging modalities include the high cost and requirement of skilled operators and equipment availability. To combat these limitations, research has been conducted to detect and monitor BCa by urinary analysis. This emerging approach remains non-invasive as it utilizes cellular components of urine to provide information on intravesical BCa [55]. Tan et al. [56] was the first group to explore this within an orthotopic xenograft model that consisted of NSG mice inoculated with low, medium, and high numbers of UM-UC-5 cells. Urinary markers of BCa (EGFR, HER2, ADAM15, and Survivin) were quantified and a tumor growth score was assigned via machine learning and principal component analysis algorithms. BLI established a correlation between these scores and tumor burden, demonstrating urinary analysis as a quantitative method for monitoring BCa. However, a plateau in scores was observed in mice inoculated with a medium to high number of BCa cells. The authors attributed this to necrosis within the center of the tumors, which affected the release of the biomarkers. This suggests that urinary analysis may not be suitable for detection or evaluation when the tumor burdens are high. The authors suggest an investigation of metastatic and invasion-specific markers to combat this. Moreover, the authors also demonstrated the use of urinary analysis to investigate the treatment efficacy. They found tumor-bearing mice treated with dacomitinib had significantly lower tumor growth scores compared to an untreated group at endpoint (*p* = 0.0002). This suggests that urinary analysis is a potential non-invasive approach to developing novel BCa therapeutics using intravesical murine models of BCa. A subsequent study demonstrated the use of urinary analysis for BCa diagnosis using activated protein kinase Cα [57]. However, its use for the longitudinal assessment of BCa progression remains unknown and requires further investigation. In conclusion, urinary analysis is a promising non-invasive alternative to the described imaging modalities when cost and availability remain an issue.

## 6. Conclusions

Current BCa treatment paradigms have limited therapeutic impact, illustrating a need for the investigation of novel therapies. Before testing the efficacy of these treatments in a clinical setting, a suitable animal model is required. This has necessitated extensive research with the aim of creating a clinically relevant intravesical murine model of BCa. Initial studies relied on sacrificing subsets of mice at varying timepoints to confirm the presence of BCa and monitor its progression. However, these few mice do not sufficiently represent what is occurring in the remainder of the cohort as it requires many experimental animals. In response to these limitations, imaging modalities such as BLI, MUI, MRI, and PET have been explored as a means of detecting the presence of BCa and longitudinally following tumor growth. These modalities allow for the repetitive, non-invasive imaging of mice, reducing the number of mice needed and optimizing the assessment of tumor response. Furthermore, they have demonstrated high sensitivity, detecting early-stage BCa prior to the onset of clinical signs, and high-resolution, imaging tumors as small as 0.4 mm^3^. Although PET requires further investigation to establish a model suitable for BCa detection, recent studies have demonstrated this to be a very promising modality. Notwithstanding the limitations of the imaging modalities discussed, many modalities have complementary strengths, which suggests that their concurrent use may provide a more sensitive and thorough analysis of BCa detection and progression in an intravesical murine model. When limitations such as cost and availability exist, urinary analysis has the potential to be used. These modalities, either independently or in a multi-modal fashion, provide information that is essential to the production of successful BCa therapies.

## Figures and Tables

**Table 1:** A summary of noninvasive imaging modalities.

Imaging Modality	Reported Advantages	Reported Limitations	Imaging Time *^ϕ^ (Minutes)	Earliest Detection Time (Days)	Detectable Tumor Size	Confirmation Rate (%)
BLI	Linearly correlated with cell quantity [10,34] and ex vivo tumor size [10]	Loss of signal overtime [35,36] Cannot detect tumor size or location [37]	12–18 [10,35,38]	4 [39]	-	91.6 [36]
MUI	MUI: Accurate detection of tumor location [40] Linearly correlated with tumor size [40] Contrast-enhanced MUI: evaluation of tumor vasculature [41]	Lack of 3-dimensional imaging [42]	MUI: ~5 [43] Contrast-enhanced MUI: ~60 [40]	4 [39]	0.4 mm^3^ [38]	87 [43]
MRI	3-dimensional imaging [44] Provide accurate tumor measurements that correlate with tumor stage and size [34,42,45]	Long examination time and high cost [42] Unable to detect tumors less than 1 mm in diameter [45]	<10 [42]	-	1.5 mm in diameter [45]	86.4 [45]
PET	Evaluates metabolic activity, identifies genomic aberrations, and protein dysregulation [46] Detects metastatic disease [47] Sensitive, quantitative, and high target selectivity [48]	Obstructed by renal excretion [47,49]	10 [50]	PET: 1 and 3 h [47,49] ImmunoPET: 72 h [48]	-	-

* Time after D-luciferin injection for BLI; ^ϕ^ Time after radiotracer injection for PET.

## Data Availability

Data contained within the article.
